# Diagnosis of osteoporotic vertebral fractures in children

**DOI:** 10.1007/s00247-018-4279-5

**Published:** 2018-11-12

**Authors:** Fawaz F. Alqahtani, Amaka C. Offiah

**Affiliations:** 1 Academic Unit of Child Health, Department of Oncology and Metabolism, Medical School, Damer Street Building, Sheffield Children’s Hospital, University of Sheffield, Western Bank, Sheffield, S10 2TH UK; 2 Department of Radiological Sciences, College of Applied Medical Sciences, Najran University, Najran, Kingdom of Saudi Arabia; 3 Radiology Department, Sheffield Children’s NHS Foundation Trust, Sheffield, UK

**Keywords:** Children, Dual energy x-ray absorptiometry, Diagnostic scoring system, Osteoporosis, Vertebral fracture, Vertebral fracture assessment

## Abstract

Osteoporosis is a generalised disorder of the skeleton with reduced bone density and abnormal bone architecture. It increases bone fragility and renders the individual susceptible to fractures. Fractures of the vertebrae are common osteoporotic fractures. Vertebral fractures may result in scoliosis or kyphosis and, because they may be clinically silent, it is imperative that vertebral fractures are diagnosed in children accurately and at an early stage, so the necessary medical care can be implemented. Traditionally, diagnosis of osteoporotic vertebral fractures has been from lateral spine radiographs; however, a small number of studies have shown that dual energy x-ray absorptiometry is comparable to radiographs for identifying vertebral fractures in children, while allowing reduced radiation exposure. The diagnosis of vertebral fractures from dual energy x-ray absorptiometry is termed vertebral fracture assessment. Existing scoring systems for vertebral fracture assessment in adults have been assessed for use in children, but there is no standardisation and observer reliability is variable. This literature review suggests the need for a semiautomated tool that (compared to the subjective and semiquantitative methods available) will allow more reliable and precise detection of vertebral fractures in children.

## Introduction

Fractures are common in childhood. About one-third of children in the United Kingdom will have at least one fracture during their childhood [1]. Osteoporotic vertebral fractures are increasingly recognised in children with either primary (e.g., osteogenesis imperfecta) [2] or secondary low bone mineral density (e.g., acute lymphoblastic leukaemia, inflammatory bowel disease and glucocorticoid use) [3, 4]. Nearly 1 in 5 children with a rheumatological condition will have a vertebral fracture [5] and rates are similar or even higher in other conditions, e.g., 16% in acute lymphoblastic leukaemia [6], up to 75% in Duchenne muscular dystrophy [7] and up to 100% in severe forms of osteogenesis imperfecta (personal experience of the senior author). Outside the context of major trauma, vertebral fractures in children indicate pathological bone fragility and precise and early diagnosis is imperative so appropriate medical care can be initiated.

Techniques to detect and analyse vertebral fractures in clinical and/or research practice include conventional radiography, computed tomography (CT), magnetic resonance imaging (MRI) and dual energy x-ray absorptiometry. Traditionally, the most common method for diagnosing vertebral fractures is x-ray, although dual energy x-ray absorptiometry has now been shown to diagnose vertebral fractures with the advantage of also determining bone mineral density [8]. Vertebral fracture assessment is the term given to the diagnosis of vertebral fractures from dual energy x-ray absorptiometry scans [9]. This technology is more or less in routine clinical use in adults, complemented by validated scoring systems [10, 11]. Conversely, vertebral fracture assessment is less widely used in children and specific paediatric scoring systems are yet to be fully validated [8, 12–15].

This review defines osteoporosis in children, summarises the factors that affect bone health, highlights diagnostic techniques in respect to vertebral fracture diagnosis and outlines the different scoring systems for recording the severity of vertebral fractures in children.

## Osteoporosis

### Definition of osteoporosis in children

According to the International Society for Clinical Densitometry (ISCD) Position Statement [16], in children without exposure to high-energy trauma or local disease, detection of one or more vertebral compression (crush) fractures (defined as a 20% reduction in vertebral body height) indicates osteoporosis. This assessment is then complemented by determining bone mineral density to give a complete evaluation of bone health. In individuals without vertebral compression (crush) fractures, osteoporosis is diagnosed based on a bone mineral density Z-score ≤-2.0 as well as a clinically significant fracture history. The latter is defined by one or more of the following:

i. Two or more long bone fractures by the age of 10 years;
ii. Three or more long bone fractures by the age of 19 years [16].

It is worth noting, therefore, that by definition, a child can neither be diagnosed with osteoporosis solely based on dual energy x-ray absorptiometry bone mineral density measurements nor before having at least one fracture.

### Factors affecting bone health

Peak bone mass, defined as the highest bone mineral content reached in an individual’s lifetime, plays a major role in determining osteoporotic fracture risk [17]. Bone strength depends on the quantity of bone present; in general, the higher the bone density, the stronger the bone. According to Golden et al. [18], factors affecting bone health may be classified as modifiable and non-modifiable. Modifiable factors such as diet, calcium, vitamin D, body weight, exercise and puberty/hormonal status [19, 20] are beyond the scope of this review, which concentrates on the non-modifiable factors of gender, age and ethnicity.

Generally, it has been observed across all ethnicities that in adults, fractures are more likely to occur in females, especially older postmenopausal women. During childhood on the other hand, boys sustain more fractures than girls at all ages. According to the study by Cooper et al. [1], which reviewed the data from 682 general practices in the United Kingdom on the General Practice Research Database, approximately 52,624 boys and 31,505 girls had one or more fractures during the 11-year follow-up period, giving a rate of 133.1/10,000 person-years. Fractures were more common in boys (incidence rate: 161.6/10,000 person-years) than in girls (incidence rate: 102.9/10,000 person-years). Other authors report that more than 50% of boys and 40% of girls have had at least one childhood fracture [21].

Boys attain a higher bone density than girls at both the lumber spine and femoral neck, but their peak values are reached at an older age [22]. In a longitudinal study conducted on 266 healthy children (136 males) ages 4-27 years (mean: 13 years), total body bone mineral density and lumbar spine and femoral neck bone mineral density were measured [23]. Males had a higher total body bone mineral density, attributed to their greater weight and lean tissue mass. In addition, boys are more likely to go through rapid rates of bone mineral accrual than girls. Given all of this, it is still uncertain whether the increase in fracture risk in boys is because of a reduction in bone mass or due to other factors such as alterations in lifestyle [24]. Thandrayen et al. [25] suggest the latter, i.e. that boys are more active than girls, thus increasing their risk of fracture.

During childhood and adolescence, bone mineral content and bone mineral density increase significantly; the increase in bone mineral density is associated with an increase in bone size. Bone mineral content and bone mineral density continue to rise across multiple skeletal sites in 16-year-old girls and 17-year-old boys, i.e. even after growth has ceased [24]. Lu et al. [23] studied the influence of age and growth on the total body, lumbar spine and femoral neck bone mineral density of 209 healthy subjects (52% boys), ages 5-27 years. There was a considerable age-dependent increase in bone mineral density in both genders at all sites, except the femoral neck in girls, which peaked at age 14 years [23]. This increase achieved the highest level around the age of 17.5 years in boys and 15.8 years in girls.

An additional study of 84,129 subjects showed that the fracture incidence increased in children between the ages of 4 and 17 years in both genders [23]. The reasons behind this high incidence of fractures in childhood are not clear. Some studies [26–28] suggest that low bone mass (caused by one or more of the modifiable and/or non-modifiable factors) may contribute.

Various studies have observed differences in bone strength across different ethnic groups. It is believed that Caucasians are more at risk of fracture than Africans and Latinos, with Asians being most at risk (owing to their relatively small bone size) [24, 25, 29–31]. Thandrayen et al. [25], in their 2009 study, compared two ethnic groups of South African children, the first being black and the second white, with both groups being of the same age and having the same gender-related distribution of fractures. They showed that white children had almost double the risk of fractures compared to black and mixed ancestry children [25]. The reason for this may be explained by the 2007 study by Kalkwarf et al. [24], which was conducted on 6– to 16-year-old females and males of varying ethnicities. At all ages, the bone mineral content and bone mineral density of the radius, hip and total body were greater for Africans compared to other ethnicities. It was discovered that African girls had more rapid pubertal development. In addition, it was identified that black girls and boys were of increased weight and height. It is worth noting that most studies have focused on Africans and Caucasians; other ethnic groups should be included in future studies.

## Assessment of bone mass and structure

Low bone mass has traditionally been considered a disease suffered by elderly people, but it is now being diagnosed in children in relatively large numbers [12]. It is generally said that if the skeletal structure of a child is weak, it is very likely to remain weak into adult life [32]. There are several noninvasive imaging techniques to assess the risk of fracture and although not all are in routine clinical use for adults and/or children, each has its own advantages and disadvantages. These methods include dual energy x-ray absorptiometry, quantitative CT, peripheral quantitative CT, high-resolution peripheral quantitative CT, quantitative ultrasound (US) and MRI.

### Dual energy x-ray absorptiometry

Dual energy x-ray absorptiometry was first introduced in the late 1980s, mostly for use in postmenopausal patients, and is now available for common use worldwide. The development of suitable algorithms allowed for paediatric dual energy x-ray absorptiometry imaging in the early 1990s. The developed algorithms detect bone/soft-tissue interfaces even in children with low bone density [33]. Although the gold standard for assessing bone mass and structure in adults is central dual energy x-ray absorptiometry of the total hip or femoral neck [34], in children, whole-body and lumbar spine (L1-L4) dual energy x-ray absorptiometry are routine and highly reproducible [35].

Bone mass is measured by dual energy x-ray absorptiometry as bone mineral content (g) or areal bone mineral density (g/cm2). Comparisons can then be made with reference values obtained from healthy children of the same age, gender and ethnic background. Results are expressed as a Z-score, the number of standard deviations the bone mineral content or bone mineral density deviates from the expected mean. The main advantages of dual energy x-ray absorptiometry are short scanning times (30-60 s), low cost, accessibility and relatively low radiation exposure (1–6 μSv) [36].

There are several limitations of dual energy x-ray absorptiometry in growing children, including inability to account for soft-tissue inhomogeneity, inclusion of the posterior elements of the vertebrae in anteroposterior imaging of the spine and dependence of the results on bone size and morphology [37]. Dual energy x-ray absorptiometry calculates areal bone mineral density by dividing the bone mineral content by the bone area without accounting for the depth of bones. Therefore, areal bone mineral density may be falsely elevated in larger bones; in other words, dual energy x-ray absorptiometry is affected by the actual size of the patient. Finally, independently assessing cortical and trabecular bone is not possible with dual energy x-ray absorptiometry [38]. The restrictions of dual energy x-ray absorptiometry hinder the assessment of bone density in infants and growing children and adolescents.

To adjust for variations in bone size and to reflect the volumetric bone mineral density, areal bone mineral density may be modified using various mathematical techniques. One technique involves the calculation of bone mineral apparent density, by dividing bone mineral content by bone volume rather than area [39]. Calculating bone mineral apparent density is relatively reliable for the hip and spine, where the shape of the bone is similar to a cylinder or cube, respectively. Other researchers have suggested that bone area and measures of body size be included in a multiple regression manner that would allow the incorporation of body size in the calculation of bone mineral content [40]. Another mathematical method, suggested by Molgaard et al. [41], involves a three-step evaluation: height for age, bone area for height, and bone mineral content for bone area and allows an assessment as to whether the child has short, narrow or light bone structure.

Although some studies have provided paediatric reference data for children and adolescents of different gender, age and ethnicity [42, 43], there is a lack of normative data for toddlers and infants (i.e. younger than 2 years old). The reasons for this are mainly due to difficulty in positioning this population appropriately, which causes movement artefact and technical challenges in measuring their small bones. Other studies have established dual energy x-ray absorptiometry reference data for infants and toddlers [44–47], but the participant numbers have been small or limited to specific populations. Kalkwarf et al. [48] provided normative bone density data of the lumber spine in 369 children ages 1 to 36 months; however, further studies are needed to demonstrate age, gender and ethnic differences in this population. Finally, the availability of dual energy x-ray absorptiometry may vary from region to region; however, this procedure is presently broadly accessible and certainly is the most widely used for bone density measurement in children.

### Quantitative computed tomography

Distinct calculations of cortical and trabecular volumetric bone mineral density can be obtained through quantitative CT using standard CT machines. Quantitative CT measures true volumetric bone mineral density (g/cm^3^) independent of body size and images to a resolution of approximately 200 μm. Quantitative CT 1) allows the calculation of bone size and geometry, both of which affect bone strength, and 2) assesses volumetric bone mineral density at axial and peripheral sites. However, quantitative CT is not a preferred option for use in children because of the high radiation dose (2.5-3.0 mSv) [36] and the absence of normative paediatric data.

### Peripheral quantitative computed tomography

Dedicated CT scanners provide an assessment of bone morphology, volumetric density and 3-D images at peripheral sites (e.g., distal radius, tibia). They independently evaluate cortical and trabecular bone with less radiation exposure than standard quantitative CT (<0.003 mSv) [36]. Cortical and trabecular measurements can be obtained from a single scan performed either at a specified but variable distance (depending on length of the bone), e.g., the 4% or 8% length of the distal radius or at a fixed site such as 10 mm from the growth plate [49, 50]. Reproducibility and positioning remain a problem and limit the use of peripheral quantitative CT in children [51].

### High-resolution peripheral quantitative computed tomography

Similar to peripheral quantitative CT, direct evaluation of bone micro architecture, an accurate measure of volumetric bone mineral density and an estimation of bone strength using finite element analysis are all possible with high-resolution peripheral quantitative CT. The advantages of high-resolution peripheral quantitative CT include high image resolution (82 μm) and low radiation exposure (<0.005 mSv) [38]. However, with limited availability of scanners, this technique is mainly used for research purposes in children. Other disadvantages are 1) the relatively long scanning time (2-3 min), which may be problematic for children, leading to movement artefact, and 2) the need for a fixed scanning site, which complicates the interpretation of longitudinal studies in growing children.

### Quantitative ultrasound

Due to dual energy x-ray absorptiometry limitations, quantitative US has been proposed as an alternative technique to measure bone properties in children. Advantages of quantitative US include its avoidance of ionizing radiation, cost effectiveness and transportability. Quantitative US depends on the attenuation of the ultrasound beam as it passes through a particular region of interest [36]. The latest instruments in the market are upgraded versions capable of giving accurate measurements for bone mineral density, in addition to reflecting parameters of bone quality and strength. Due to the large amount of soft tissue and muscles at axial sites, quantitative US can only be applied to appendicular bones including phalanges, radius, calcaneus, patella and tibia. In general, studies of quantitative US have shown good intra- and inter-operator reliability with coefficient of variation ranging from 0.3 to 3.7% and 0.3 to 1.2%, respectively. The reliability of quantitative US in children and adolescents has been assessed in several studies [52–55] and although it may potentially [41–49] be useful for bone density assessment, its true utility from a clinical perspective has not been adequately addressed in children and it remains a research tool.

### Magnetic resonance imaging

Like quantitative US, MRI has the advantage of avoiding the use of ionizing radiation. However, there is a lack of reference scanning protocols and normative data for bone density assessment in children and its use is still limited to research studies. Further drawbacks of MRI are the long scanning time (acquisition time of up to 10-20 min) [56, 57], requirement for specialised coils and the environment of the scanner room (isolated from carers/parents, noisy), which may not be child-friendly [58]. Despite these disadvantages, some studies have suggested MRI is a promising technique to evaluate bone properties in children [59–62]. However, before widespread clinical use of MRI to determine bone mass in children, technical and software advancement is required to improve the reproducibility of measurements.

## Imaging of vertebral fractures

Methods that may be used for detecting and analysing vertebral fractures in clinical practice are conventional radiography, dual energy x-ray absorptiometry, MRI and CT. Differences among these imaging techniques relate to radiation dose, accessibility, cost and patient convenience. These various factors are summarised in Table 1.

**Table 1:** Imaging modalities for detecting vertebral fractures in children

Modality	Spatial resolution (μm)	Effective radiation dose for whole-spine scanning (μSv)	Scan time (min)	Approximate cost (single scan - including cost of reporting in British £) [63]
Conventional radiography	100×100	233 [64]	˂1	37
Computed tomography	600×600	10,000 [12]	˂1	74-100
Magnetic resonance imaging	234×234×500	None	10-30	120-163
Dual energy x-ray absorptiometry	350×350	3 [65]	˂1	58

### Conventional radiography

Currently, radiography is the most common imaging tool for detecting vertebral fracture in children and adolescents. It is frequently the initial imaging investigation of choice for back pain when skeletal disease/vertebral pathology is suspected, since the resolution is excellent. However, there is significant radiation exposure (232.7 μSv) [64], equal to 12 months’ background radiation [66].

Assessing the height and shape of the T4-L4 vertebral bodies from lateral thoracic and lumbar spine radiographs is the standard method [9]. Vertebral levels T1 to T3 are not routinely assessed because of the difficulty in their visualisation due to the summation caused by overlying structures such as intrathoracic organs and the patient’s shoulders. The normal physiological wedging that may be seen of the mid-thoracic vertebrae (T5 to T7) should not be mistaken for fracture.

The accuracy of vertebral fracture diagnosis in children from conventional radiographs has been evaluated in several studies (Table 2) [4, 12, 13, 64, 67, 68]. In general, vertebral levels T7–L4 are highly visible (visibility ranging from 88 to 99.8%), whereas visibility is more limited in the upper part of the thoracic spine (T4- T7) for the reasons given above and because of often poor image quality and poor patient positioning. Inter- and intra-observer agreement for vertebral readability range between kappa of 0.33 to 0.98 and 0.43 to 0.76, respectively, and for fracture diagnosis range between kappa of 0.43 to 0.66 and 0.52 to 0.76, respectively. Unfortunately, despite the radiation dose, conventional radiographs remain the gold standard imaging modality for diagnosing vertebral fractures in children, mainly due to convenience and availability.

**Table 2:** Summary of more recent published studies for vertebral fracture diagnosis in children

Reference	Number of patients	Median age (years)	Scoring system	Imaging modality	Number of observers	Sensitivity and specificity	Agreement level (Kappa)
Mayranpaa et al. 2007 [12]	65	12.1	Genant semiquantitative technique	Radiographs and dual energy x-ray absorptiometry	1 radiologist 1 orthopaedic spine surgeon	Not available	Radiographs Inter-observer 0.98 Dual energy x-ray absorptiometry Inter-observer 0.34
Halton et al. 2009 [4]	186	5.3	Genant semiquantitative technique	Radiographs	2 radiologists	Not available	Inter-observer for fracture defined as Grade 1, 2 or 3 0.44 (95% confidence interval 0.28–0.59) Inter-observer for fracture defined as Grade 2 and 3 0.66 (95% confidence interval 0.46–0.87)
Siminoski et al. 2014 [67]	186	9.6	Genant semiquantitative technique	Radiographs	3 radiologists	Not available	Readability* Inter-observer 1) *Vertebral level* 0.33 to 0.50 2) *Patient level* 0.29 to 0.46 Intra-observer 1) *Vertebral level* 0.43 to 0.64 2) *Patient level* 0.41 to 0.61 Vertebral fracture: Inter-observer 1) *Vertebral level* 0.45 to 0.54 2) *Patient level* 0.43 to 0.48 Intra-observer 1) *Vertebral level* 0.52 to 0.72 2) *Patient level* 0.52 to 0.76
Diacinti et al. 2015 [13]	58	7.0	Genant semiquantitative technique	Dual energy x-ray absorptiome try	2 radiologists	Sensitivity 96% Specificity 100%	Inter-observer 1) *Vertebral level* 0.81 (95% confidence interval 0.76–0.86) 2) *Patient level* 0.96 (95% confidence interval 0.89–1.03)
Kyriakou et al. 2015 [14]	165	13.4	Genant semiquantitative technique	Dual energy x-ray absorptiometry	2 non-radiologists	Sensitivity 75% Specificity 98%	Readability Inter-observer 1) *Vertebral level* 0.73 (95% confidence interval, 0.68–0.73) 2) *Patient level* 0.66 (95% confidence interval, 0.56, 0.77) Vertebral fracture: Inter-observer 1) *Vertebral level* 0.85 (95% confidence interval, 0.79–0.91) 2) *Patient level* 0.78 (95% confidence interval, 0.66–0.87)
Adiotomre et al. 2017 [64]	250	11.5	Simplified algorithm-based qualitative technique	Radiographs and dual energy x-ray absorptiometry	3 radiologists	Dual energy x-ray absorptiometry Sensitivity 70% (95% confidence interval 58–82%) Specificity 97% (95% confidence interval 94–100%) Radiographs Sensitivity 84% (95% confidence interval 0.70–0.99) Specificity 72% (95% confidence interval 0.47–0.97)	Dual energy x-ray absorptiometry Inter-observer 0.37 Intra-observer 0.63 Radiographs Inter-observer 0.42 Intra-observer 0.62
Crabtree et al. 2017 [15]	80	12.0	Genant semiquantitative technique	Dual energy x-ray absorptiometry	1 paediatric radiologist 2 paediatricians	Sensitivity 1) *Vertebral level* 66% 2) *Patient level* 82% Specificity 1) *Vertebral level* 95% 2) *Patient level* 78%	Inter-observer 1) *Vertebral level* 0.63 (95% confidence interval 0.56–0.69) 2) *Patient level* 0.60 (95% confidence interval 0.42–0.77)
Crabtree et al. 2017 [15]	80	12.0	Morphometric analysis	Dual energy x-ray absorptiometry	1 paediatric radiologist 2 clinical scientists 1 radiographer	Sensitivity 1) *Vertebral level* 79% 2) *Patient level* 43% Specificity 1) *Vertebral level* 71% 2) *Patient level* 97%	Inter-observer 1) *Vertebral level* 0.32 (95% confidence interval 0.26–0.38) 2) *Patient level* 0.41 (95% confidence interval 0.24–0.59)
Alqahtani et al. 2017 [68]	137	12.0	Morphometric analysis using SpineAnalyzer	Radiographs	1 paediatric radiologist 2 radiographers 2 medical Students	Sensitivity 18% (95% confidence interval 0.14–0.22) Specificity 97% (95% confidence interval 0.97–0.98)	Inter-observer 0.05–0.47 Intra-observer using intraclass correlation coefficient 0.25–0.61

*Readability refers to the rate of radiographs/dual energy x-ray absorptiometry scans that were of sufficient quality to be interpretable and was calculated by dividing the number of readable vertebrae for each vertebral level/patient level by each observer (intra-observer) and/or by all observers (inter-observer)

**Fig. 1:**
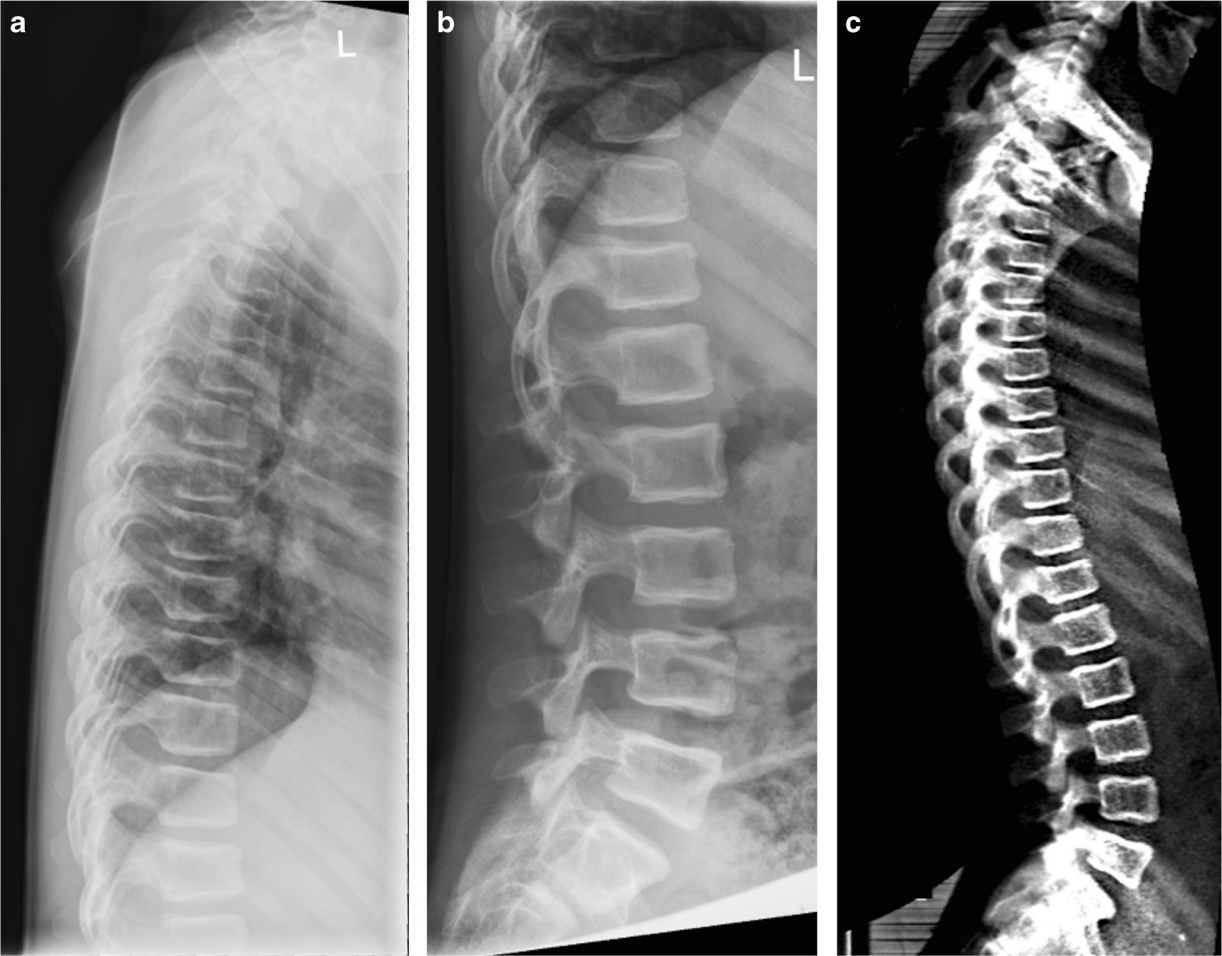
An 11-year-old boy with osteogenesis imperfecta. **a-c** Lateral thoracic (**a**) and lumbar spine (**b**) radiographs are juxtaposed to a lateral spine dual energy x-ray absorptiometry scan (**c**) performed on the same day. The image quality of (**c**) is non-inferior to (**a**) and (**b**), with the advantage of being a single image

It is essential to have good quality spine radiographs to precisely evaluate vertebral fractures and associated deformities. In order to generate good-quality lateral images, the spine should be as parallel as possible to the radiographic table [69]. Generally, a 100-cm focus-to-film distance should be maintained. For thoracic radiographs, the beam is centred at T7, and for lumbar radiographs, it’s centred at L3. With younger children, it is possible to perform the whole spine on a single radiograph where the central beam points to T12. For accurate lateral views, position patients on their left side with flexed knees and hips [13]. Figure 1 shows lateral thoracic and lumbar spine radiographs of an ideally positioned patient.

### Dual energy x-ray absorptiometry

Despite the limitations of dual energy x-ray absorptiometry for assessing bone density/predicting fracture risk in children, as discussed above, dual energy x-ray absorptiometry is now considered a significant tool for assessing and monitoring bone health [70]. The development of machines that allow lateral imaging has expanded the role of dual energy x-ray absorptiometry beyond assessing bone strength to include the assessment of bone morphometry/vertebral fractures diagnosis, termed vertebral fracture assessment.

Vertebral fracture assessment has exciting potential. It is easily and rapidly applicable during bone mineral density measurement, thus enhancing the management of osteoporotic patients [65], obviates the need for spine radiographs and affords point-of-service convenience for the patient because the imaging is performed at the same visit and at the same time as the dual energy x-ray absorptiometry for bone mineral density measurement [64, 66]. Perhaps the most significant advantage of vertebral fracture assessment is the radiation dose savings that it allows; for instance, a dose as low as 3 μSv has been reported for dual energy x-ray absorptiometry by some authors [65, 71]. Another advantage is the ability of dual energy x-ray absorptiometry to acquire the whole lateral spine (of larger patients) in a single image; whereas with radiography, the thoracic and lumber spine require two separate images (Fig. 1).

Many recent studies have assessed the reliability of vertebral fracture assessment in adults and shown good performance with sensitivity and specificity ranging from 62 to 97% and 94 to 99%, respectively, and observer agreement (kappa score) ranging between 0.24 and 0.98 [72–79].

Mayranpaa et al. [12], in their 2007 study, showed that vertebral fracture assessment produces uncertain results in children with low bone mineral density, and they argued that improvements in the image quality of lateral dual energy x-ray absorptiometry and in scoring systems for vertebral fracture assessment were necessary before this approach could be used reliably in children. In contrast, a recent larger study showed similar sensitivity (78% and 72%) and specificity (84% and 72%) for dual energy x-ray absorptiometry and radiographs, respectively, indicating that vertebral fracture assessment is as good as conventional radiography for diagnosis of vertebral fractures in children [64]. This study is the only one to address visualisation of non-vertebral body fractures (spondylolysis/spondylolisthesis) and the effects of spinal rods and patient positioning. The study showed that the quality of the two modalities was comparable and, in fact, superior for dual energy x-ray absorptiometry in the presence of spinal rods [64]. The study by Crabtree et al. [15] also demonstrated that dual energy x-ray absorptiometry is comparable to radiographs for detecting moderate and severe vertebral fractures with sensitivity and specificity of 81.3% and 99.3%, respectively. It is worth noting the poor diagnostic accuracy of mild vertebral fractures in children, irrespective of imaging modality, possibly related to poor distinction from normal variants and non-fracture pathology [64].

To summarise, in the past, vertebral fracture assessment was found to be inappropriate for paediatric use due to poor image quality, with numerous false-positive findings, an inability to identify vertebrae in small children and a failure to distinguish physiological changes in morphology. However, results of recent studies (Table 2) indicate that vertebral fracture assessment is a promising technique for diagnosing vertebral fractures in children [12, 14, 15, 64, 67].

Previously, lateral spine dual energy x-ray absorptiometry scans were obtained with the patient in a decubitus position (lying on their side), but with newer machines, the patient remains in a supine position (lying on their back) and rather than the patient moving, the machine’s c-arm rotates to obtain a lateral image of the whole spine.

For decubitus views, the child lies on his/her side on the scanning table. Arms should be kept away from the area to be scanned and should be at a right angle (90^o^) to the chest. The knees should be flexed upwards towards the chest, so that the spine is parallel to the scanning table. Foam pads may be used to obtain and maintain the required position. Subsequently, the process of acquiring the dual energy x-ray absorptiometry scans should be conducted as recommended by the manufacturer. The child should be reminded to stay still throughout the examination [80].

For supine views, the child should lie on the scanning table in the supine position with his/her arms raised above their head. The patient’s spine should be positioned in the centre of the scanning area and both knees should be raised upwards using foam pads to straighten up the base of the spine.

### Computed tomography and magnetic resonance imaging

CT and MRI are variably used for diagnosis of vertebral fracture in children. CT is the most reliable and accurate method for vertebral morphology evaluation when an acute traumatic fracture is suspected. Disadvantages include a high radiation dose penalty (approximately 10,000 μSv for whole spine scanning, equal to 3 years’ background radiation) [12], generally reduced availability of machines and relative expense.

MRI has the advantage of not utilising ionising radiation and helps to differentiate the underlying cause of vertebral fractures other than osteoporosis (particularly malignancy), but such scans take a relatively long time and are more costly than other modalities.

Neither CT nor MRI are indicated for routine monitoring/diagnosis of osteoporotic vertebral fractures in children.

### Biplanar X-ray imaging

The biplanar x-ray imaging system (EOS imaging, Paris, France) is a relatively new imaging solution meeting the specific needs of musculoskeletal imaging. The system produces high-quality images of the whole body, including the whole spine, at lower radiation dose than radiographs (for lateral spine, mean entrance surface dose was 0.37 mGy compared with 2.03 mGy for radiographs) and it has the ability to generate 3-D images from simultaneous anteroposterior and lateral 2-D images of the whole body [81]. EOS scanning time ranges from 10 to 20 s for a full body scan and 4–6 s for the spine (depending on the patient’s height). EOS plays a major role in the diagnosis and follow-up of patients with adolescent idiopathic scoliosis [82, 83] and has been used to diagnose vertebral fractures in 200 patients older than 50 years in whom it was compared to vertebral fracture assessment (dual energy x-ray absorptiometry device: QDR 4500; Hologic, Bedford, MA) [83]. The sensitivity, specificity and negative predictive values for EOS were 79.7%, 91.6% and 99%, respectively. Inter-observer agreement between two independent readers was very good for EOS (kappa score=0.89), higher than for vertebral fracture assessment (kappa score=0.67). We are not aware of any study that has compared EOS with radiographs and/or vertebral fracture assessment for the diagnosis of vertebral fractures in children; however, the results in adults suggest that EOS is a good diagnostic tool for the diagnosis of vertebral fractures. Therefore, it is also likely to be beneficial in children with advantages of high image quality, low dose and rapid acquisition time. Further research studies are needed to assess diagnostic accuracy of vertebral fracture in children using EOS.

## Scoring systems for vertebral fractures

When using any classification system, the normal slight curve of the lower lumbar vertebrae and anatomical changes such as wedging of mid-thoracic and thoracolumbar vertebrae should be borne in mind. Baseline and serial radiographs should be compared with one another to document improvement/deterioration in prevalent (i.e. previously identified) vertebral fractures and to detect incident (i.e. new) vertebral fractures [84].

Quantitative morphometric definitions [85–88] and semiquantitative assessments of vertebral fractures, including methods by Smith [89] and Kleerekoper [90] have been introduced for adults; however, this review focuses only on methods that have been used in children.

### Subjective visual assessment

The most extensively employed method for assessing vertebral fractures is visual assessment of radiographs [91]. Qualitative visual assessment is helpful when performed by experts capable of disregarding abnormal appearances that have nothing to do with the osteoporotic fracture. However, due to the subjectivity of the technique, observer reliability is low with inter- and intra-observer kappa scores of 0.47 and 0.62, respectively [92]. In other words, the findings of visual assessment greatly depend on the competency of the reader. For this reason, visual assessment is not recommended for epidemiological studies or therapeutic trials.

### Genant’s semiquantitative assessment

**Fig. 2:**
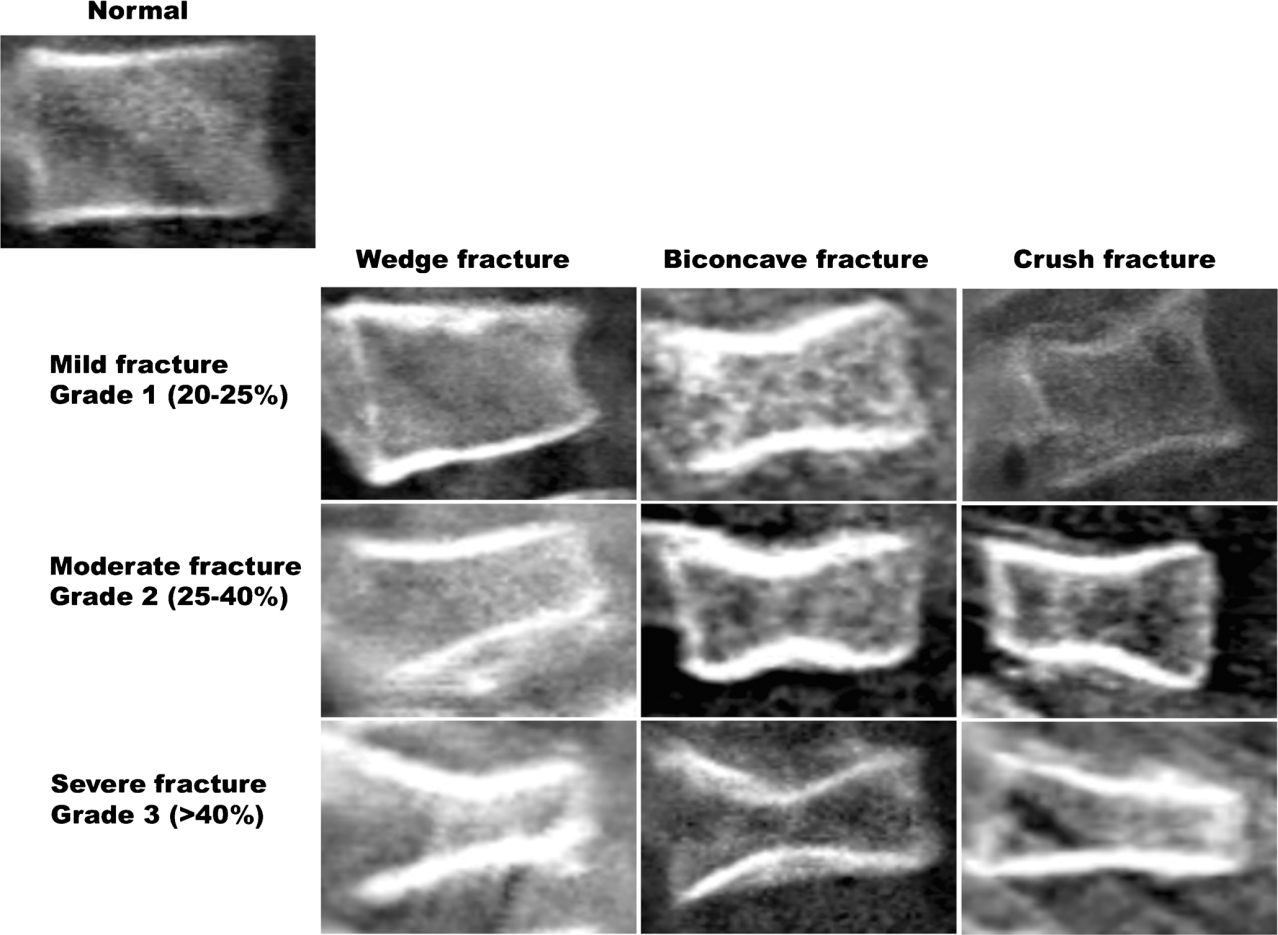
Selected lateral spine dual energy x-ray absorptiometry scans from a series of patients demonstrate the semiquantitative visual grading system of Genant et al. [10]

This semiquantitative grading system was developed in 1993 based on independent analysis of the spine radiographs of 57 postmenopausal women (ages 65-75 years) by three observers [10]. Assessment is made of vertebral shape (crush, concave or wedge) and reduction in posterior, middle and/or anterior vertebral height. Grade 0 indicates no fracture (normal) with a height reduction of less than 20%, Grade 1 indicates a minimal fracture with a height reduction in the range of 20-25%, Grade 2 indicates a moderate fracture with a height reduction in the range of 25-40% and Grade 3 indicates a severe fracture with a height reduction of above 40% (Fig. 2). Although this method was developed for and is the standard tool for diagnosing vertebral fractures in adults, researchers have begun to assess its use for diagnosing vertebral fractures in children [4, 13, 14, 67]. These paediatric studies demonstrate inter- and intra-observer reliability ranging from kappa score=0.29 to 0.98 and 0.41 to 0.63, respectively, and sensitivity and specificity ranging from 66 to 95% and 78 to 100%, respectively. The variability between the different studies for observer reliability and sensitivity/specificity may reflect limitations of the quantitative method such as false-positive identification of non-fracture deformities, disparity in fracture prevalence and severity within the study cohorts and misdiagnosis of mild endplate fractures (i.e. mild height loss may be physiological rather than pathological).

The benefits of Genant’s semiquantitative method include its convenience, being less complicated than quantitative methods, improved consistency when compared to qualitative methods and the fact that it can be used by both experts and beginners with acceptable reproducibility and precision [93, 94]*.* However, although reduced compared to subjective visual assessment, the experience and competency of the reader still greatly influences its implementation. A further drawback of this method is that deformation of shape is not taken into consideration while making the evaluation [84]. The fracture is detected by observing the reduction in vertebral height or area, but radiological features related to vertebral endplate abnormality are not considered [11].

### Algorithm-based qualitative method

The algorithm-based qualitative method is not based on reduced vertebral height alone; this system provides clear guidelines for the evaluation of alterations in the vertebral endplates, helpful in detecting osteoporotic fractures in adults [95]. It has been pointed out that although three grades of severity are present, just as in Genant’s system, there is no lower limit for Grade 1 fractures (Grade 1 ≤25%, Grade 2 >25%, and Grade 3 >40%) [11]. Nevertheless, the algorithm-based qualitative approach offers the advantage of addressing potential sources of false-positive detection of vertebral fracture such as “deep inferior” and “step-like” endplates [95].The algorithm serves as a basic guideline for qualitative identification and differentiation of vertebral fracture, non-fracture deformity and normal variant. However, observers need to be fully trained in the application of the method, and the algorithm should be applied with recourse to reference notes on differential diagnoses.

A recent study was the first to analyse the clinical utility of an adapted version of the algorithm-based qualitative approach from radiographs and dual energy x-ray absorptiometry in children [8]. Development of the scoring system was in two phases: modification of algorithm-based qualitative and simplification of the modified algorithm-based qualitative system. The researchers showed slight to good inter-observer agreement in 50 patients by both modified algorithm-based qualitative (kappa score=0.27 to 0.49) and simplified algorithm-based qualitative (kappa score=0.31 to 0.45) and moderate intra-observer for simplified algorithm-based qualitative (kappa score=0.45 to 0.56). All observers subjectively found simplified algorithm-based qualitative easier and less time-consuming, which makes it more appealing for clinical and research use compared to modified algorithm-based qualitative for scoring vertebral fractures in children [8].

Although the algorithm-based qualitative technique is promising as a semi-objective means of classifying osteoporotic vertebral fractures in adults, there is only limited research into this technique in children. Further studies are required to assess whether the simplified algorithm-based qualitative method is sufficiently reliable to identify and differentiate fracture from non-fracture deformities in children.

### Koerber’s technique

Recently, a new scoring system was introduced for assessing spine morphology in children with osteogenesis imperfecta (developed using 268 lateral spine radiographs of 95 patients) [96]. The assessment is based on three criteria: vertebral compression, thoracolumbar kyphosis and deformity type, with a scale of 1 to 5 to defining severity (1=no need for therapy and 5=extremely severe). To record all possible combinations of the three parameters, the authors developed a more detailed severity score system ranging from 1 to 138. The authors state that this evaluation will benefit patients in clinics; however, it seems that this method is limited by being relatively time-consuming (a trained reader needs from 5 to 8 min to define the category and severity scores) [96].

### Semiautomated techniques

**Fig. 3:**
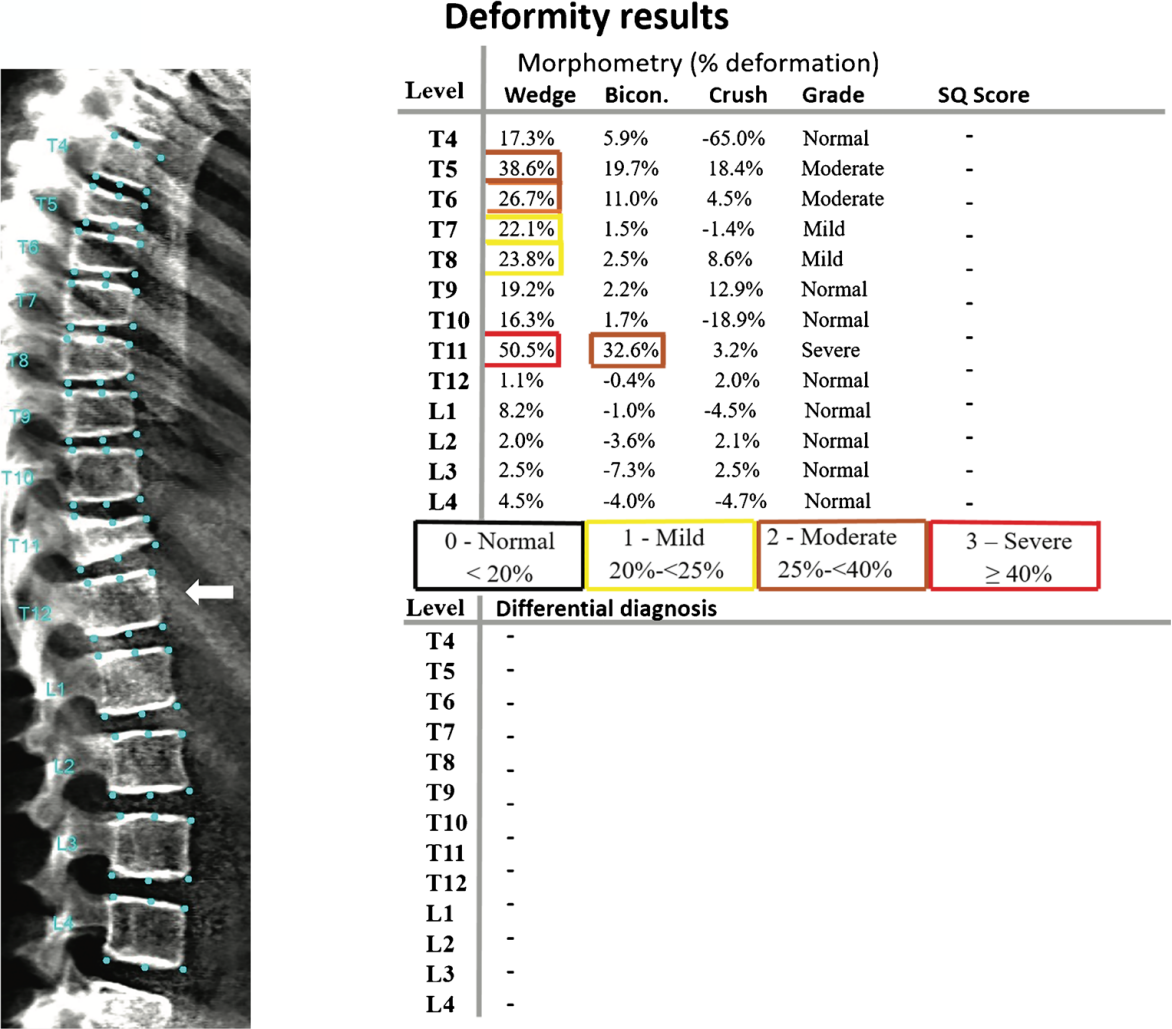
A 14-year-old girl with osteogenesis imperfecta. Lateral spine dual energy x-ray absorptiometry scan illustrates positioning of points used to outline the vertebral bodies between T4 and L4 using the SpineAnalyzer program. SpineAnalyzer identified a severe fracture at T11, moderate fractures at T5 and T6 and mild fractures at T7 and T8. The *arrow* points to the T12 vertebral body (lowest vertebral body associated with a rib). SQ Semiquantitative, *Bicon*. Biconcave

Semiautomated quantitative vertebral morphometry techniques typically employ model-based shape recognition technology to define the shape of all vertebrae between T4 and L4 inclusive (Fig. 3) [97].

The procedure (termed 6-point morphometric analysis) begins with a manual indication of the estimated centre of each vertebra from T4 to L4. The software then mechanically identifies and marks the standard positions for six-point morphometry measurements. The operator may move these points with the help of the software for improved accuracy. From these six points, anterior (H^a^), middle (H^m^) and posterior (H^p^) vertebral heights are automatically determined by the software. Then, the (H^a^_:_ H^p^), (H^m^_:_ H^p^), (H^p^_:_ H^p+1^) and (H^p^_:_ H^p-1^) height ratios are calculated (^+1^ and ^−1^ indicate the vertebrae immediately above and below the vertebra of interest). Each vertebral body is then classified according to its height ratios based on the Genant classification (Grades 0 to 3).

SpineAnalyzer software (Optasia Medical Ltd., Cheadle, UK) provides a quick and easy method for identifying and reporting vertebral deformities from radiographs and other x-ray-based technology. A recent study concluded that SpineAnalyzer is a reliable and ideal system for measuring vertebral height and identifying vertebral fracture from dual energy x-ray absorptiometry scans in adults, with significant observer agreement (ranging from 96 to 98.6%) using the Genant semiquantitative method [98]. Although studies have used the semiautomated quantitative software to diagnose fractures in adults, as far as we are aware, only two studies have used this semiautomated six-point software technique to diagnose vertebral fractures in children [15, 68].

The study by Alqahtani et al. [68] is the largest morphometric analysis study in children using SpineAnalyzer; the study assessed 137 lateral spine radiographs of children between ages 5 and 15 years. Inter- and intra-observer agreement, overall sensitivity and specificity are shown in Table 2. Another study by Crabtree et al. [15] demonstrated poor observer agreement of morphometric analysis for vertebral fracture diagnosis in 80 children (Table 2).

The results of these two papers suggest that the diagnostic accuracy of semiautomated systems/morphometric analysis is not sufficiently high to allow their routine clinical use in children. Therefore, training of current software programmes on paediatric images or development of paediatric specific software and reference values is required.

## Summary

Identifying vertebral fracture is central to the diagnosis of osteoporosis in children. Imaging methods used to detect and analyse vertebral fractures in clinical and/or research practice include conventional radiographs, dual energy x-ray absorptiometry (vertebral fracture assessment), CT and MRI. While vertebral fracture assessment is routine in adults, identifying vertebral fracture in children is still mostly from radiographs. However, recent pediatric studies have shown that dual energy x-ray absorptiometry vertebral fracture assessment has similar sensitivity and specificity to radiographs with lower radiation dose; therefore, dual energy x-ray absorptiometry should be considered for vertebral fracture diagnosis in children, when feasible. It is likely that EOS will have an increasing role.

There is no agreed standardised method for diagnosing vertebral fracture in children and it is difficult to be certain of the validity of mild fractures.

## Conclusion

There is no reliable method to assess vertebral fractures in children. This situation may be improved by the development of a software tool for semiautomated vertebral fracture assessment. Such a tool should be specifically designed for paediatric use and encompass normal physiological variation, which almost certainly accounts for some observer variability in diagnosing vertebral fractures in this population.

## References

[1] Cooper C, Dennison EM, Leufkens HG (2004). Epidemiology of childhood fractures in Britain: a study using the general practice research database. J Bone Miner Res.

[2] Bishop N (2010). Characterising and treating osteogenesis imperfecta. Early Hum Dev.

[3] Mäkitie O (2013). Causes, mechanisms and management of paediatric osteoporosis. Nat Rev Rheumatol.

[4] Halton J, Gaboury I, Grant R et al (2009) Advanced vertebral fracture among newly diagnosed children with acute lymphoblastic leukemia: results of the Canadian steroid-associated osteoporosis in the pediatric population (STOPP) research program. J Bone Miner Res 24:1326–1334 10.1359/jbmr.090202 PMC3890351 19210218

[5] Nakhla M, Scuccimarri R, Duffy KN (2009). Prevalence of vertebral fractures in children with chronic rheumatic diseases at risk for osteopenia. J Pediatr.

[6] Alos N, Grant RM, Ramsay T (2012). High incidence of vertebral fractures in children with acute lymphoblastic leukemia 12 months after the initiation of therapy. J Clin Oncol.

[7] Bothwell JE, Gordon KE, Dooley JM (2003). Vertebral fractures in boys with Duchenne muscular dystrophy. Clin Pediatr (Phila).

[8] Adiotomre E, Summers L, Allison A (2016). Diagnosis of vertebral fractures in children: is a simplified algorithm-based qualitative technique reliable?. Pediatr Radiol.

[9] Maricic M (2014). Use of DXA-based technology for detection and assessment of risk of vertebral fracture in rheumatology practice. Curr Rheumatol Rep.

[10] Genant HK, Wu CY, Vankuijk C, Nevitt MC (1993). Vertebral fracture assessment using a semiquantitative technique. J Bone Miner Res.

[11] Jiang G, Eastell R, Barrington NA, Ferrar L (2004). Comparison of methods for the visual identification of prevalent vertebral fracture in osteoporosis. Osteoporos Int.

[12] Mayranpaa MK, Helenius I, Valta H (2007). Bone densitometry in the diagnosis of vertebral fractures in children: accuracy of vertebral fracture assessment. Bone.

[13] Diacinti D, Pisani D, D'Avanzo M (2015). Reliability of vertebral fractures assessment (VFA) in children with osteogenesis imperfecta. Calcif Tissue Int.

[14] Kyriakou A, Shepherd S, Mason A, Faisal Ahmed S (2015). A critical appraisal of vertebral fracture assessment in paediatrics. Bone.

[15] Crabtree NJ, Chapman S, Högler W (2017). Vertebral fractures assessment in children: evaluation of DXA imaging versus conventional spine radiography. Bone.

[16] Bishop N, Arundel P, Clark E (2014). Fracture prediction and the definition of osteoporosis in children and adolescents: the ISCD 2013 pediatric official positions. J Clin Densitom.

[17] Bonjour JP, Theintz G, Law F (1994). Peak bone mass. Osteoporos Int.

[18] Golden NH, Abrams SA, Committee on Nutrition (2014). Optimizing bone health in children and adolescents. Pediatrics.

[19] Ondrak KS, Morgan DW (2007). Physical activity, calcium intake and bone health in children and adolescents. Sports Med.

[20] van Leeuwen J, Koes BW, Paulis WD, Middelkoop M (2017). Differences in bone mineral density between normal-weight children and children with overweight and obesity: a systematic review and meta-analysis. Obes Rev.

[21] Amin S, Melton LJ, Achenbach SJ (2013). A distal forearm fracture in childhood is associated with an increased risk for future fragility fractures in adult men, but not women. J Bone Miner Res.

[22] Bianchi ML, Sawyer AJ, Bachrach LK (2016) Rationale for bone health assessment in childhood and adolescence. Bone health assessment in pediatrics. Springer, Cham, pp 1–21

[23] Lu PW, Briody JN, Ogle GD (1994). Bone-mineral density of total-body, spine, and femoral-neck in children and young-adults - a cross-sectional and longitudinal-study. J Bone Miner Res.

[24] Kalkwarf HJ, Zemel BS, Gilsanz V (2007). The bone mineral density in childhood study: bone mineral content and density according to age, sex, and race. J Clin Endocrinol Metab.

[25] Thandrayen K, Norris SA, Pettifor JM (2009) Fracture rates in urban South African children of different ethnic origins: the birth to twenty cohort. Osteoporos Int 20:47–52 10.1007/s00198-008-0627-x PMC2859163 18465189

[26] Clark EM, Tobias JH, Ness AR (2006). Association between bone density and fractures in children: a systematic review and meta-analysis. Pediatrics.

[27] Ma D, Jones G (2003). The association between bone mineral density, metacarpal morphometry, and upper limb fractures in children: a population-based case-control study. J Clin Endocrinol Metab.

[28] Schalamon J, Singer G, Schwantzer G, Nietosvaara Y (2004). Quantitative ultrasound assessment in children with fractures. J Bone Miner Res.

[29] Cummings SR, Melton LJ (2002). Epidemiology and outcomes of osteoporotic fractures. Lancet.

[30] Bachrach LK, Hastie T, Wang M-C (1999). Bone mineral acquisition in healthy Asian, Hispanic, black, and Caucasian youth: a longitudinal study. J Clin Endocrinol Metab.

[31] Li JY, Specker BL, Ho ML, Tsang RC (1989). Bone mineral content in black and white children 1 to 6 years of age: early appearance of race and sex differences. Am J Dis Child.

[32] Ma NS, Gordon CM (2012). Pediatric osteoporosis: where are we now?. J Pediatr.

[33] Specker B, Schoenau E (2006). Quantitative bone analysis in children: current methods and recommendations. J Pediatr.

[34] Mauck KF, Clarke BL (2006). Diagnosis, screening, prevention, and treatment of osteoporosis. Mayo Clin Proc.

[35] Crabtree NJ, Arabi A, Bachrach LK (2014). Dual-energy X-ray absorptiometry interpretation and reporting in children and adolescents: the revised 2013 ISCD pediatric official positions. J Clin Densitom.

[36] Adams JE (2016). Bone densitometry in children. Semin Musculoskelet Radiol.

[37] Wren TA, Gilsanz V (2006). Assessing bone mass in children and adolescents. Curr Osteoporos Rep.

[38] Digby MG, Bishop NJ, Paggiosi MA, Offiah AC (2016). HR-pQCT: a non-invasive ‘biopsy’ to assess bone structure and strength. Arch Dis Child Educ Pract Ed.

[39] Carter DR, Bouxsein ML, Marcus R (1992). New approaches for interpreting projected bone densitometry data. J Bone Miner Res.

[40] Prentice A, Parsons TJ, Cole TJ (1994). Uncritical use of bone-mineral density in absorptiometry may lead to size-related artifacts in the identification of bone-mineral determinants. Am J Clin Nutr.

[41] Molgaard C, Thomsen BL, Prentice A (1997). Whole body bone mineral content in healthy children and adolescents. Arch Dis Child.

[42] Ward KA, Ashby RL, Roberts SA (2007). UK reference data for the Hologic QDR discovery dual-energy x ray absorptiometry scanner in healthy children and young adults aged 6-17 years. Arch Dis Child.

[43] Crabtree NJ, Shaw NJ, Bishop NJ (2017). Amalgamated reference data for size-adjusted bone densitometry measurements in 3598 children and young adults-the ALPHABET study. J Bone Miner Res.

[44] Specker BL, Beck A, Kalkwarf H, Ho M (1997). Randomized trial of varying mineral intake on total body bone mineral accretion during the first year of life. Pediatrics.

[45] Butte NF, Hopkinson JM, Wong WW (2000). Body composition during the first 2 years of life: an updated reference. Pediatr Res.

[46] Ay L, Jaddoe VW, Hofman A et al (2011) Foetal and postnatal growth and bone mass at 6 months: the generation R study. Clin Endocrinol 74:181–190 10.1111/j.1365-2265.2010.03918.x 21050252

[47] Gallo S, Vanstone CA, Weiler HA (2012). Normative data for bone mass in healthy term infants from birth to 1 year of age. J Osteoporos.

[48] Kalkwarf HJ, Zemel BS, Yolton K, Heubi JE (2013). Bone mineral content and density of the lumbar spine of infants and toddlers: influence of age, sex, race, growth, and human milk feeding. J Bone Miner Res.

[49] Neu CM, Manz F, Rauch F (2001). Bone densities and bone size at the distal radius in healthy children and adolescents: a study using peripheral quantitative computed tomography. Bone.

[50] Wang Q, Alen M, Nicholson P (2005). Growth patterns at distal radius and tibial shaft in pubertal girls: a 2-year longitudinal study. J Bone Miner Res.

[51] Saraff V, Hoegler W (2015). Osteoporosis in children: diagnosis and management. Eur J Endocrinol.

[52] Pereira-da-Silva L, Costa A, Pereira L (2011). Early high calcium and phosphorus intake by parenteral nutrition prevents short-term bone strength decline in preterm infants. J Pediatr Gastroenterol Nutr.

[53] Tomlinson C, McDevitt H, Ahmed SF, White MP (2006). Longitudinal changes in bone health as assessed by the speed of sound in very low birth weight preterm infants. J Pediatr.

[54] Roggero P, Giannì ML, Orsi A (2007). Postnatal “speed of sound” decline in preterm infants: an exploratory study. J Pediatr Gastroenterol Nutr.

[55] Wang KC, Wang KC, Amirabadi A (2014). Evidence-based outcomes on diagnostic accuracy of quantitative ultrasound for assessment of pediatric osteoporosis—a systematic review. Pediatr Radiol.

[56] Krug R, Burghardt AJ, Majumdar S, Link TM (2010). High-resolution imaging techniques for the assessment of osteoporosis. Radiol Clin N Am.

[57] Link TM, Majumdar S, Grampp S (1999). Imaging of trabecular bone structure in osteoporosis. Eur Radiol.

[58] Ward KA, Link TM, Adams JE (2016) Tools for measuring bone in children and adolescents. Bone health assessment in pediatrics. Springer, Cham, pp 23–52

[59] Bass SL, Saxon L, Daly RM (2002). The effect of mechanical loading on the size and shape of bone in pre-, peri-, and postpubertal girls: a study in tennis players. J Bone Miner Res.

[60] McKay HA, Sievänen H, Petit MA (2004). Application of magnetic resonance imaging to evaluation of femoral neck structure in growing girls. J Clin Densitom.

[61] Heinonen A, McKay H, Whittall K (2001). Muscle cross-sectional area is associated with specific site of bone in prepubertal girls: a quantitative magnetic resonance imaging study. Bone.

[62] Duncan CS, Blimkie CJ, Kemp A (2002). Mid-femur geometry and biomechanical properties in 15-to 18-yr-old female athletes. Med Sci Sports Exerc.

[63] Improvement NHS (2018) National tariff payment system 2017/18 and 2018/19. https://improvement.nhs.uk/resources/national-tariff-1719/. Accessed 13 February 2018

[64] Adiotomre E, Summers L, Allison A (2017). Diagnostic accuracy of DXA compared to conventional spine radiographs for the detection of vertebral fractures in children. Eur Radiol.

[65] Damilakis J, Adams JE, Guglielmi G, Link TM (2010). Radiation exposure in X-ray-based imaging techniques used in osteoporosis. Eur Radiol.

[66] Lewiecki EM, Laster AJ (2006). Clinical review: clinical applications of vertebral fracture assessment by dual-energy x-ray absorptiometry. J Clin Endocrinol Metab.

[67] Siminoski K, Lentle B, Matzinger MA (2014). Observer agreement in pediatric semiquantitative vertebral fracture diagnosis. Pediatr Radiol.

[68] Alqahtani FF, Messina F, Kruger E (2017). Evaluation of a semi-automated software program for the identification of vertebral fractures in children. Clin Radiol.

[69] Link TM, Guglielmi G, van Kuijk C, Adams JE (2005). Radiologic assessment of osteoporotic vertebral fractures: diagnostic and prognostic implications. Eur Radiol.

[70] Cummings SR, Bates D, Black DM (2002). Clinical use of bone densitometry: scientific review. JAMA.

[71] Vokes T, Bachman D, Baim S (2006). Vertebral fracture assessment: the 2005 ISCD official positions. J Clin Densitom.

[72] Hospers IC, van der Laan JG, Zeebregts CJ (2009). Vertebral fracture assessment in supine position: comparison by using conventional semiquantitative radiography and visual radiography. Radiology.

[73] Binkley N, Krueger D, Gangnon R (2005). Lateral vertebral assessment: a valuable technique to detect clinically significant vertebral fractures. Osteoporos Int.

[74] Fuerst T, Wu C, Genant HK (2009). Evaluation of vertebral fracture assessment by dual X-ray absorptiometry in a multicenter setting. Osteoporos Int.

[75] Buehring B, Krueger D, Checovich M (2010). Vertebral fracture assessment: impact of instrument and reader. Osteoporos Int.

[76] Vokes TJ, Dixon LB, Favus MJ (2003). Clinical utility of dual-energy vertebral assessment (DVA). Osteoporos Int.

[77] Schousboe JT, DeBold CR (2006). Reliability and accuracy of vertebral fracture assessment with densitometry compared to radiography in clinical practice. Osteoporos Int.

[78] Rea JA, Chen MB, Li J (1999). Morphometric X-ray absorptiometry and morphometric radiography of the spine: a comparison of analysis precision in normal and osteoporotic subjects. Osteoporos Int.

[79] Ferrar L, Jiang G, Eastell R, Peel NF (2003). Visual identification of vertebral fractures in osteoporosis using morphometric X-ray absorptiometry. J Bone Miner Res.

[80] Crabtree NJ, Kent K (2016) Acquisition of DXA in children and adolescents. Bone health assessment in pediatrics. Springer, Cham, pp 89–114

[81] Wade R, Yang H, McKenna C (2013). A systematic review of the clinical effectiveness of EOS 2D/3D X-ray imaging system. Eur Spine J.

[82] Rehm J, Germann T, Akbar M (2017). 3D-modeling of the spine using EOS imaging system: inter-reader reproducibility and reliability. PLoS One.

[83] Briot K, Fechtenbaum J, Etcheto A (2015). Diagnosis of vertebral fractures using a low-dose biplanar imaging system. Osteoporos Int.

[84] Damilakis J, Maris TG, Karantanas AH (2007). An update on the assessment of osteoporosis using radiologic techniques. Eur Radiol.

[84] Melton LJ, Kan SH, Frye MA (1989). Epidemiology of vertebral fractures in women. Am J Epidemiol.

[86] Eastell R, Cedel SL, Wahner HW (1991). Classification of vertebral fractures. J Bone Miner Res.

[87] Minne HW, Leidig G, Wuster C et al (1988) A newly developed spine deformity index (SDI) to quantitate vertebral crush fractures in patients with osteoporosis. Bone Miner 3:335–349 2852512

[88] McCloskey EV, Spector TD, Eyres KS (1993). The assessment of vertebral deformity: a method for use in population studies and clinical trials. Osteoporos Int.

[89] Smith RW, Eyler WR, Mellinger RC (1960). On the incidence of senile osteoporosis. Ann Intern Med.

[90] Kleerekoper M, Parfitt AM, Ellis BI (1984) Measurement of vertebral fracture rates in osteoporosis. In: Christiansen C, Arnaud CD, Nordin BEC, Parfitt AM, Peck WA, Riggs BL (Eds.) Osteoporosis: Proceedings of the Copenhagen International Symposium on Osteoporosis. Osteopress, Copenhagen, pp 103–109

[91] Grados F, Fechtenbaum J, Flipon E (2009). Radiographic methods for evaluating osteoporotic vertebral fractures. Joint Bone Spine.

[92] Jensen GF, McNair P, Boesen J, Hegedüs V (1984). Validity in diagnosing osteoporosis. Observer variation in interpreting spinal radiographs. Eur J Radiol.

[93] Genant HK, Jergas M, Palermo L (1996). Comparison of semiquantitative visual and quantitative morphometric assessment of prevalent and incident vertebral fractures in osteoporosis. J Bone Miner Res.

[94] Panda A, Das CJ, Baruah U (2014). Imaging of vertebral fractures. Indian J Endocrinol Metab.

[95] Ferrar L, Jiang G, Adams J, Eastell R (2005). Identification of vertebral fractures: an update. Osteoporos Int.

[96] Koerber F, Uphoff US, Koerber S (2012). Introduction of a new standardized assessment score of spine morphology in osteogenesis imperfecta. Rofo.

[96] Kim YM, Demissie S, Eisenberg R (2011). Intra-and inter-reader reliability of semi-automated quantitative morphometry measurements and vertebral fracture assessment using lateral scout views from computed tomography. Osteoporos Int.

[98] Birch C, Knapp K, Hopkins S (2015). SpineAnalyzer™ is an accurate and precise method of vertebral fracture detection and classification on dual-energy lateral vertebral assessment scans. Radiography.

